# Tribute to Our Reviewers 2024

**DOI:** 10.1002/deo2.70098

**Published:** 2025-03-15

The Editorial Board of *DEN Open* takes this opportunity to acknowledge with particular gratitude the following reviewers who have provided their valuable time and expertise in the evaluation of manuscripts submitted to *DEN Open* during the period of 1 January 2024 to 31 December 2024.

Takao Itoi

Editor‐in‐Chief

*DEN Open*

Nobutsugu Abe

Kyoichi Adachi

Yoichi Akazawa

Teppei Akimoto

Shintaro Akiyama

Ikuo Aoyama

Taiki Aoyama

Reiko Ashida

Shinya Ashizuka

Maki Ayaki

Rafael Castro

Shannon Chan

Li‐Chun Chang

Hideyuki Chiba

Eduardo De Moura

Akira Dobashi

Osamu Dohi

Shinpei Doi

Alanna Ebigbo

Mitsuru Esaki

Yuki Fujii

Nao Fujimori

Ai Fujimoto

Toshio Fujisawa

Akashi Fujita

Yuji Fujita

Mitsuharu Fukasawa

Nobuhiko Fukuba

Shuichi Fukuda

Hayato Fukui

Shusei Fukunaga

Yoshihiro Furuichi

Kenichi Goda

Osamu Goto

Ryunosuke Hakuta

Natalie Halvorsen

Kenta Hamada

Yasuhiko Hamada

Kazuo Hara

Ryo Harada

Minami Hashimoto

Shinichi Hashimoto

Hisashi Hidaka

Kazutoshi Higuchi

Takuma Higurashi

Takuto Hikichi

Makoto Hinokuchi

Hiroto Hiraga

Kingo Hirasawa

Takashi Hirose

Takashi Hisabe

Yusuke Horiuchi

Kinichi Hotta

Ryoji Ichijima

Katsuro Ichimasa

Eikichi Ihara

Toshiro Iizuka

Yuichiro Ikebuchi

Eriko Ikeda

Hisatomo Ikehara

Atsushi Imagawa

Hajime Imai

Ken Inoue

Tadahisa Inoue

Fumiaki Ishibashi

Yusuke Ishida

Kazunaga Ishigaki

Ryu Ishihara

Naoki Ishii

Hirotoshi Ishiwatari

Ken Ito

Masanori Ito

Nobuhito Ito

Sayo Ito

Masahiro Itonaga

Hiroyoshi Iwagami

Naoto Iwai

Masaya Iwamuro

Takuji Iwashita

Keisuke Iwata

Taro Iwatsubo

Ryuhei Jinushi

Tomohiro Kadota

Hideki Kamada

Ken Kamata

Takashi Kanesaka

Atsushi Kanno

Yoshihide Kanno

Hiromitsu Kanzaki

Chikatoshi Katada

Motohiko Kato

Tsunetaka Kato

Noriyuki Kawami

Seiji Kawano

Noboru Kawata

Daisuke Kikuchi

Toshifumi Kin

Satoshi Kinoshita

Koh Kitagawa

Katsuya Kitamura

Yoko Kitamura

Masanori Kobayashi

Yousuke Kobayashi

Shinya Kodashima

Takehiko Koga

Hirofumi Kogure

Yoriaki Komeda

Yoshiyasu Kono

Shinsuke Koshita

Kensuke Kubota

Takahiro Kudo

Yu‐Ting Kuo

Chika Kusano

Takamichi Kuwahara

Toshio Kuwai

Yasuharu Maeda

Mai Makiguchi

Akinori Maruta

Hirotsugu Maruyama

Atsuhiro Masuda

Saburo Matsubara

Akihisa Matsuda

Takahisa Matsuda

Hiroshi Matsumoto

Kazuyuki Matsumoto

Tomoaki Matsumura

Yukitoshi Matsunami

Ryo Matsuoka

Noriko Matsuura

Kosuke Minaga

Tatsunori Minamide

Yosuke Minoda

Masashi Misawa

Shuichi Miyamoto

Masaki Miyaoka

Kurato Miyazaki

Kazuhiro Mizukami

Suguru Mizuno

Douglas Motomura

Takashi Murakami

Tatsuro Murano

Kazumasa Nagai

Yasuaki Nagami

Itaru Naitoh

Kazunari Nakahara

So Nakaji

Keiichiro Nakajo

Jun Nakamura

Masanao Nakamura

Atsushi Nakayama

Ken Namikawa

Daiki Nemoto

Tsutomu Nishida

Noriko Nishiyama

Toshihiro Nishizawa

Shuzo Nomura

Satoru Nonaka

Yohei Ogata

Ken Ohata

Akihisa Ohno

Eizaburo Ohno

Masayuki Ohta

Kensei Ohtsu

Kazuo Ohtsuka

Tomohiko Ohya

Shiro Oka

Yutaka Okagawa

Takeshi Okamoto

Mitsuru Okuno

Nozomi Okuno

Teppei Omori

Shunsuke Omoto

Yoichiro Ono

Takumi Onoyama

Shozo Osera

Kazuhiro Ota

Koji Otani

Akira Ouchi

Mohan Ramchandani

Ryota Sagami

Tomotaka Saito

Arata Sakai

Taku Sakamoto

Yasuhisa Sakata

Fumisato Sasaki

Yu Sasaki

Chiaki Sato

Chiko Sato

Hiroki Sato

Tatsuya Sato

Yoshinori Sato

Masau Sekiguchi

Satoki Shichijo

Minoru Shigekawa

Yuto Shimamura

Masaaki Shimatani

Takaya Shimura

Satoshi Shinozaki

Hideyuki Shiomi

Kazuo Shiotsuki

Hironari Shiwaku

Koichi Soga

Noriaki Sugawara

Mitsushige Sugimoto

Shinya Sugimoto

Harutoshi Sugiyama

Keijiro Sunada

Haruhisa Suzuki

Rei Suzuki

Sho Suzuki

Jun Takada

Kazunori Takada

Naminatsu Takahara

Koji Takahashi

Kosuke Takahashi

Hiroyuki Takamaru

Yusuke Takasaki

Ayaka Takasu

Yusaku Takatori

Hidetoshi Takedatsu

Mamoru Takenaka

Kotaro Takeshita

Tetsuya Takikawa

Naoto Tamai

Yuzuru Tamaru

Takashi Tamaura

Fumio Tanaka

Masaki Tanaka

Takehiro Tanaka

Yuki Tanisaka

Yusuke Taniyama

Tomoaki Tashima

Tetsuya Tatsuta

Keiichi Tominaga

Yuri Tomita

Takeshi Tomoda

Ryosuke Tonozuka

Yosuke Toya

Haruka Toyonaga

Takayoshi Tsuchiya

Yosuke Tsuji

Koshiro Tsutsumi

Kunihisha Uchita

Takeshi Uozumi

Kunihiko Wakamura

Tosiyuki Wakatsuki

Pu Wang

Kenta Watanabe

Reiko Yamada

Takeshi Yamada

Daisuke Yamaguchi

Tomohiro Yamaguchi

Akira Yamamiya

Keiko Yamamoto

Yoichi Yamamoto

Yoshinobu Yamamoto

Kentaro Yamao

Yasushi Yamasaki

Takeshi Yamashina

Shunich Yanai

Kei Yane

Takeshi Yasuda

Yoshihiro Yokoyama

Masao Yoshida

Naohiro Yoshida

Naohisa Yoshida

Shinji Yoshii

Toshiyuki Yoshio

Shinichiro Yoshioka

Tetsuya Yoshizaki

Andee Dzulkarnaen Zakaria

